# Strong family history and early onset of schizophrenia: about 2 families in Northern Nigeria

**DOI:** 10.11604/pamj.2016.24.282.7568

**Published:** 2016-07-28

**Authors:** Folorunsho Tajudeen Nuhu, Edwin Ehi Eseigbe, Baba Awoye Issa, Michael Omeiza Gomina

**Affiliations:** 1 Federal Neuropsychiatric Hospital, Barnawa Kaduna, Nigeria; 2 Department of Paediatrics, Ahmadu Bello University Teaching Hospital, Zaria Nigeria; 3 Department of Behavioural Science, University of Ilorin Teaching Hospital, Ilorin Nigeria

**Keywords:** Schizophrenia, genetic loading, age of onset, outcome

## Abstract

Schizophrenia is a highly heritable psychotic disorder and high genetic loading is associated with early onset of the disease. The outcome of schizophrenia has also been linked with the age of onset as well as the presence of family history of the disease. Therefore families with patients with early onset Schizophrenia are subpopulations for genetic studies. We present 2 families with heavy genetic loading who have adolescents with schizophrenia.

## Introduction

Studies have reported that early-onset schizophrenia, defined by manifestation of psychotic symptoms before 18 years of age, occurs in 4% of all schizophrenia cases [1]. It has also been shown that this early onset cases are familial variant of the adult-onset schizophrenia with strong genetic liability [2]. Because early-onset cases show clinical, cognitive, genetic and neurobiologic continuity with adult-onset schizophrenia but with enduring clinical morbidity and psychosocial disability [3], it is necessary to identify families with early onset disease. Doing so might be able to identify cases with poor psychosocial performance and appropriate intervention done to ameliorate it. These case series describe cases of early onset schizophrenia in two families, thus buttressing a strong genetic predisposition.

## Patient and observation

**CASE 1:** The mother is a known patient with Schizophrenia presently in remission on medications. The father has a personality that is submissive and dependent who goes extra-mile to please his wife and relatives. However he has not met diagnostic criteria for any major mental disorder. All their 3 daughters are schizophrenic (with onset at ages 10, 12 and 15 years) Miss SB is 13 years old, diagnosed at age 10. She was brought to the hospital on account of physical and verbal aggression especially towards immediate elder sister claiming she wanted to kill her, hearing voices of unseen people calling her name, talking to herself, speaking incomprehensible language and neglecting personal hygiene. She also ran away from home and hid herself in the bush even at night. Other symptoms included laughing without obvious reason and self injurious behaviour-cutting self with razor blade. The above symptoms started 8 months before presentation. Mental State Examination revealed a restless young girl sitting on bare floor, talking alone with poor eye contact. Her speech was incoherent. She exhibited regressed behaviour -urinated on self while being examined and attempted playing with urine. Physical Examination showed slightly big for age with ‘gigantic’ facial look Miss EB is 15 years old, diagnosed at the age of 12 years. She presented with social withdrawal, stripping self naked even in the presence of strangers, crying unnecessarily when younger sister beats her, neglecting self care, declining school performance, gesticulating as if communicating with unseen people and asking questions as if she was a small baby such as “dad will you buy aero-plane for me”. Mental State Examination revealed an unkempt adolescent, laughing fatuously, covering her face with her hands, most of the time murmuring to herself and giving irrelevant answers to questions. She worked out of the consulting room and refused to come back, spitting indiscriminately in the waiting room. Physical Examination was normal Miss BB is an 18 year old fifth year secondary school student who presented at the age of 15 years with undue suspiciousness-family members plotting to kill her, hearing parents discussing their plans to get rid of her, refusing food. She was neglecting her personal hygiene, restless and verbally aggressive at home especially towards her mother. She slept poorly and would not attend school. On Mental State Examination, we found a fairly kempt young lady with slurred but coherent speech. She exhibited prominent persecutory delusions and auditory hallucination with voices discussing her in the third person. Her cognitive function was good but she had no insight into her illness. Physical Examination did not reveal any abnormality. In this first family; all the children were delivered spontaneously with vertex presentation. Pregnancies were termed and the mother was in a good physical health condition. The first patient had neonatal jaundice described as mild which was treated only by exposing the child to sun. Jaundice was said to have cleared completely within 3 days. All the children attained normal developmental milestones and they all had good social interaction before the onsets of symptoms. They performed above average at school before illness and the 3^rd^ patient was still performing well at the time of writing this case series. All of them improved on antipsychotic drugs but only the oldest achieved complete resolution of symptoms for up to 6 months.

**CASE 2:** The father is a 39 year old trader who was diagnosed with schizophrenia at the age of 33 years and he is presently in remission but still on medication. Patient's aunt (father's immediate younger sister) is 35 years old and was diagnosed as schizophrenic at age 26, also on treatment. She has had several relapses but presently in remission. Master PG, 15 year old forth year secondary school student was diagnosed at the age of 12 years as schizophrenic. He presented with; excessive searching for ‘imaginary items’, checking the clock repeatedly with no apparent reason, loss of concentration in school, declining performance, irrational behaviour-defecating in the bathroom instead of toilet and urinating on self. He was also isolating himself from people as well as refusing to talk and eat. He also maintained odd and same position for a long time, staring blankly in to space and occasionally he talked out of context. Other symptoms included refusal to take his bath even when prompted and refusal to go to school claiming they were on holiday. Pregnancy, birth, neonatal and childhood history and development milestones were normal. His performance in school before illness was described as above average. Mental State findings included; unkempt, stinking (urine) adolescent maintaining same position, with waxy flexibility, occasional negativism, mutism, fixing gaze/staring. During the assessment he was hiding himself under the chair and urinated and defecated on the floor. On the 2^nd^ day of admission a bedside assessment showed a good cognitive function including arithmetic and language. However he continued to exhibit disorganized and hallucinatory behaviours. For all the 4 patients, various differential diagnoses were considered and ruled out-Pervasive developmental disorders, ADHD, School refusal, *Mental retardation (*clinically). They were all treated with antipsychotic medications. However only the oldest of them responded well to treatment and achieved complete remission. In those with very early onset schizophrenia (VEOS), different classes of antipsychotic drugs at maximum tolerable doses for at least 6 weeks were administered as well as modified electro-convulsive therapy. In spite of this, they still had some residual symptoms.

## Discussion

These cases highlight the early onset of the disorder in the 4 patients with strong genetic predisposition. Three of the patients have the VEOS (onset before the age of 13 years) according to the classification based on the age of onset [4, 5] while only one was diagnosed at the age of 15 years. In these cases both sexes were reported. Previous researchers have indicated that early onset schizophrenia (EOS) is associated with greater genetic loading [5, 6]. Uncorrelated monozygotic and dizygotic concordances of 88.7% and 22.9%, respectively, have also been reported [7]. In addition, family studies have shown that child and adolescent onset schizophrenia carries a greater familial risk than adult onset [8] and 20% of child and adolescent onset schizophrenia had at least one primary relative with schizophrenia and 50% had a first-degree relative with psychosis [9]. In our subjects, both families had more than one relative with schizophrenia. In the first family, when each patient is considered as a case there were 3 other members suffering from schizophrenia. In the second family, the patient's father and aunt are currently receiving treatment for schizophrenia. It is also noteworthy that negative symptoms predict a family history of schizophrenia [10, 11] and indeed 2 of our patients had some negative symptoms. In addition, the poor response to treatment in 3 of the patients is in keeping with previous report that early onset of schizophrenia could be a risk factor for poor outcome [12] and this poor response can also be explained in relation to the negative symptoms and presence of family history [12] Schizophrenia, a heterogeneous disorder, has been linked to multiple genes which have been hypothesized to be the aetiological basis of the disease [13] as well as some chromosomal deletions. For instance, **ventrocardofacial** Syndrome (VCFS)-a ventrodeletion of chromosome “22q”was implicated. Patients with VCFS have 2% risk of Schizophrenia Vs 0.2% in normal population [14] and there is a risk of early onset Schizophrenia in VCFS [15]. In the National Institute of Mental Health study of childhood onset schizophrenia, 5 out of 47 cases had previously undetected cytogenetic abnormality; 6.3% of which had VCFS, 1 had Turner syndrome and 1 with a balanced translocation of chromosomes 1 and 7 [16]. There is an association between CAG/CTG trinucleotide expansion and childhood schizophrenia [17]. In addition a single nucleotide polymorphism such as estrogen receptor alpha (ESRα) is said to play an important role in age of onset of schizophrenia [18].

## Conclusion

These case series support previous findings outside Nigeria that early onset schizophrenia is associated with strong genetic predisposition. Previous genetic studies have reported several different chromosomal abnormalities in different environments. Could these be the same, similar or different in Nigerian patients. This report will serve as a basis for conducting such genetic studies in Nigeria.
